# Survey of tick species and molecular detection of selected tick-borne pathogens in Yanbian, China[Fn FN1]

**DOI:** 10.1051/parasite/2022039

**Published:** 2022-07-21

**Authors:** Jixu Li, Shuang Zhang, Wanfeng Liang, Shaowei Zhao, Zhenyu Wang, Hang Li, Bingyi Yang, Zhen Zhang, Jialin Li, Lijun Jia

**Affiliations:** 1 Engineering Research Center of North-East Cold Region Beef Cattle Science & Technology Innovation, Ministry of Education, Yanbian University No. 977 Park Road Yanji 133002 PR China; 2 Yanbian Center for Disease Control and Prevention Yanji 133001 PR China; 3 Department of Preventive Medicine, Medical College of Yanbian University Yanji 133002 PR China

**Keywords:** Yanbian, Tick, *Rickettsia*, Severe fever thrombocytopenia syndrome virus, *Theileria*

## Abstract

Ticks and tick-borne diseases pose a significant threat to public health. In this study, we aimed to determine the tick species distribution and pathogens carried by ticks in Yanbian, China. A total of 2673 questing ticks were collected from eight counties and cities in Yanbian and were morphologically identified. The presence of *Candidatus* Rickettsia tarasevichiae (CRT), spotted fever group *Rickettsia* (SFGR), severe fever thrombocytopenia syndrome virus (SFTSV), *Theileria*, and other pathogens was confirmed using polymerase chain reaction (PCR) and real-time quantitative PCR assays, followed by phylogenetic and genotypic analyses. According to the morphological identification, the tick species in Yanbian consisted of *Haemaphysalis longicornis*, *Ixodes persulcatus*, *Dermacentor silvarum*, *H. japonica*, and *H. concinna*. In *H. longicornis*, CRT, SFGR, SFTSV and *Theileria orientalis* were detected, while CRT, SFGR, and SFTSV were detected in *I. persulcatus*, *H. japonica*, and *D. silvarum*. Only SFTSV was detected in *H. concinna*. Mixed infection with CRT and SFTSV was observed in *I. persulcatus* and *H. japonica*. The gene sequences of all tested pathogens exhibited 95.7%–100% identity with the corresponding sequences deposited in GenBank. Phylogenetic analysis showed that different SFGR and SFTSV genotypes were closely related to the Korean strains. This study is the first to describe the genetic diversity of SFGR *Candidatus* Rickettsia longicornii in *H. longicornis* in Yanbian, China, using the *ompA*, *ompB*, *sca4*, and *rrs* genes. These results provide epidemiological data to support the prevention and control of ticks and tick-borne diseases in the border areas of China, North Korea, and Russia.

## Abstract (fr)

Les tiques et les maladies transmises par les tiques constituent une menace importante pour la santé publique. Dans cette étude, nous avons cherché à déterminer la distribution des espèces et les agents pathogènes portés par les tiques à Yanbian, en Chine. Un total de 2 673 tiques errantes ont été collectées dans huit comtés et villes de Yanbian et identifiées morphologiquement. La présence de *Candidatus* Rickettsia tarasevichiae (CRT), de *Rickettsia* du groupe de la fièvre boutonneuse (SFGR), du virus du syndrome de la fièvre thrombocytopénique sévère (SFTSV), de *Theileria* et d’autres agents pathogènes a été confirmée à l’aide d’une réaction en chaîne par polymérase (PCR) et de PCR quantitative en temps réel, suivies par des analyses phylogénétiques et génotypiques. Selon leur identification morphologique, les espèces de tiques à Yanbian se composaient de *Haemaphysalis longicornis*, *Ixodes persulcatus*, *Dermacentor silvarum*, *H. japonica* et *H. concinna*. Chez *H. longicornis*, CRT, SFGR, SFTSV et *Theileria orientalis* ont été détectés, tandis que CRT, SFGR et SFTSV ont été détectés chez *I. persulcatus*, *H. japonica* et *D. silvarum*. Seul le SFTSV a été détecté chez *H. concinna*. Une infection mixte par CRT et SFTSV a été observée chez *I. persulcatus* et *H. japonica*. Les séquences des gènes de tous les agents pathogènes testés présentaient une identité de 95,7 % à 100 % avec les séquences correspondantes déposées dans GenBank. L’analyse phylogénétique a montré que différents génotypes SFGR et SFTSV étaient étroitement liés aux souches coréennes. Cette étude est la première à décrire la diversité génétique de SFGR *Candidatus* Rickettsia longicornii chez *H. longicornis* à Yanbian, en Chine, en utilisant les gènes *ompA*, *ompB*, *sca4* et *rrs*. Ces résultats fournissent des données épidémiologiques pour soutenir la prévention et le contrôle des tiques et des maladies transmises par les tiques dans les zones frontalières de la Chine, de la Corée du Nord et de la Russie.

## Introduction

Ticks act as vectors for many pathogens and are widespread in nature [16, 25]. Ticks include the families Ixodidae, Argasidae, Deinocrotonidae, and Nuttalliellidae [2, 17]. A tick bite penetrates the skin of the host, which can result in dermatitis, ulcers, or secondary infection. The presence of large numbers of ticks in livestock not only damages the affected area, but also results in anemia and reduced development, yield, and quality of the livestock; sometimes, infestation even results in death. More and more studies have clarified the ability of ticks to carry and transmit a variety of disease-causing pathogens [18], including viruses, protozoa, and bacteria [24]. Ticks and tick-borne diseases cause substantial harm to human health and animal husbandry, and have become an important public health problem worldwide [14, 19].

China covers a vast area and includes regions with dramatically different natural conditions. There are obvious differences in the distribution of ticks between the southern and the northern regions of the country, and tick-borne diseases occasionally occur. Yanbian is located at 41°59′47″ – 44°30′ 42″ North latitude, 127°27′43″ – 131°18′33″ East longitude, and is bordered by North Korea and Russia. This region, approximately covering 43,329.34 km^2^, is largely covered in forest and has a mid-temperate humid monsoon climate. It is rich in biological resources and provides suitable conditions for the development and reproduction of ticks. However, the species distribution of ticks and tick-borne pathogens in the border area of China, Russia, and North Korea is unclear. Therefore, we systematically examined the species distribution and pathogens carried by ticks in Yanbian to provide a scientific basis to support the prevention and control of ticks and tick-borne diseases in Yanbian.

## Materials and methods

### Ethics

All experimental procedures in animals were conducted following the Ethical Principles in Animal Research issued by Yanbian University (approval number: 20180301).

### Sample collection

**Figure 1:**
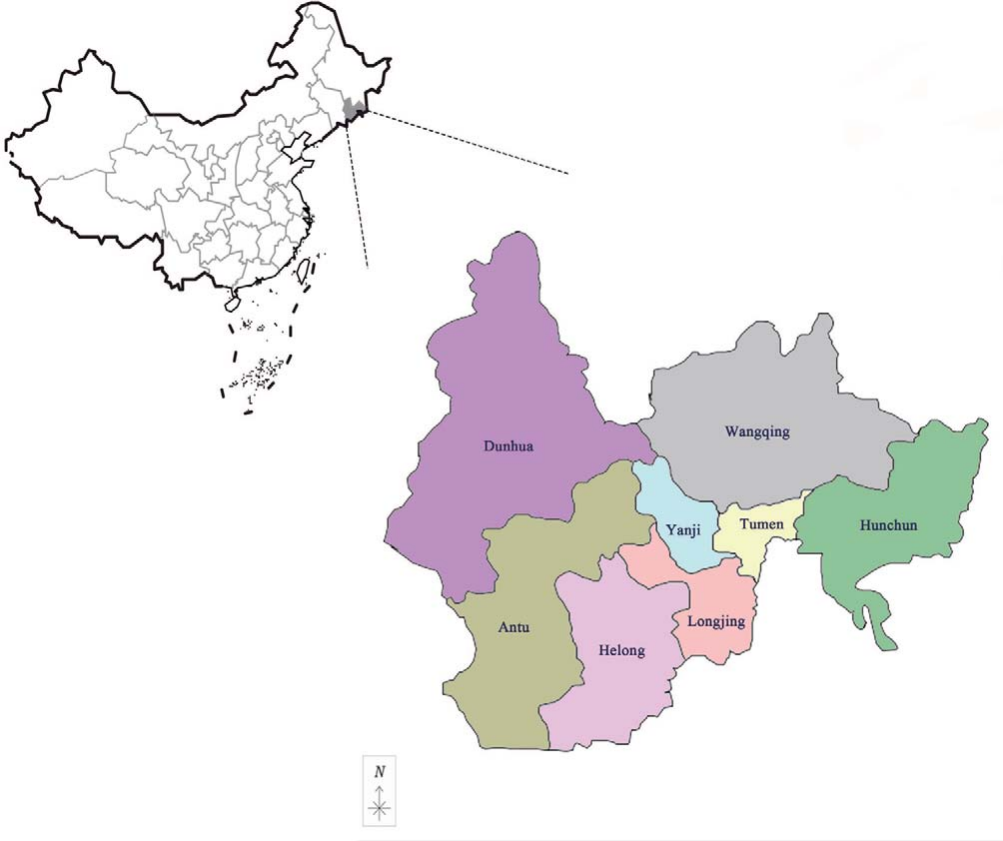
Map of sampling districts in Yanbian, Jilin, China. The different colors represent the various districts sampled in this study.

A total of 2673 unattached adult ticks were collected from Hunchun, Tumen, Yanji, Dunhua, Helong, Longjing, Wangqing, and Antu in Yanbian, China (Fig. 1), during sunny mornings from April to August 2019, using the dragging-flagging method. A white gauze mesh, 2 m long and 1 m wide, was completely spread out on the grass and dragged slowly over the grass to capture ticks. Ticks attached to the gauze mesh were then transferred to the collection tube by tweezers, and the related information, such as collection time and location, were marked in detail. The ticks were stored live in water in the refrigerator at 4 °C. The date and place of collection was recorded for each sample.

### Tick classification and nucleic acid extraction

The collected ticks were classified morphologically based on the “Classification and Identification of Important Medical Insects of China” [13] and “Ticks of Japan, Korea, and the Ryukyu Islands” [21] by examining the specimens under a microscope (OLYMPUS CX23, Japan).

The ticks were grouped according to species and region. Each tick was washed three times in normal saline solution, placed in a 1.5-mL centrifuge tube, and 600-μL phosphate-buffered saline were added. The ticks were then ground with a tissue breaker (TissueLyserII; Qiagen, Germany), followed by centrifugation at 1300× *g* for 1 min. Approximately 200 μL of the supernatant were collected to extract the nucleic acid. RNA was extracted from samples using an RNA extraction kit (Suzhou Tianlong Science and Technology Co., Ltd., Suzhou, China), and used to synthesize cDNA using a reverse transcription kit (Tiangen, Beijing, China).

### Pathogen detection

The *Candidatus* Rickettsia tarasevichiae (CRT) *ompA* and *17-kDa* genes were amplified as described by Jia *et al.* [4]. The spotted fever group *Rickettsia* (SFGR) *Candidatus* Rickettsia longicornii *ompA*, *ompB*, *sca4*, *rrs* genes were detected as described by Jiang *et al.* [3]. The severe fever thrombocytopenia syndrome virus (SFTSV) Small, Medium, and Large gene segments were detected as described by Liu *et al.* [10]. The *Theileria orientalis* MPSP gene was amplified as described by Ota *et al.* [15] and the *Theileria sinensis* MPSP gene was detected as described by Liu *et al.* [8]. The primers used in this study are listed in Table 1.

**Table 1:** Primer sequences used for the gene amplification of different pathogens.

Pathogen gene	Primer name	Sequence (5′–3′)	Annealing temperature (°C)	Fragment size (bp)	Reference
CRT *ompA*	Rr190.70p	ATGGCGAATATTTCTCCAAAA	60	346	Jia *et al.* [4]
	Rr190.602n	AGTGCAGCATTCGCTCCCCCT			
	190.70-38s1	AAAACCG CTTTATTCACC	58		
	190.602-384r1	GGCAAC AAGTTACCTCCT			
CRT *17kDa*	17K3	GCTTTACAAAATTCTAAAAACCATATA	50	395	Jia *et al.* [4]
	17K5	TGTCTATCAATTCACAACTTGCC			
	17KD113s1	ATTGTCCGTCAGGTTGGC	52		
	17KD408r1	CGGGCGGTATGAATAAGC			
SFGR *Candidatus* Rickettsia longicornii *ompA*	H-LompA-F	TTTAATTGATTTAATTTTTATTAAGGTTTACATATGGCG	60	647	Jiang *et al.* [3]
	H-LompA-R	GTCTTGACAGTTATTATACCTCCTCCAT			
SFGR *Candidatus* Rickettsia longicornii *ompB*	H-LompB-F1	GTTCAGCTATGGGTGCTGCTATACAG	63	1203	Jiang *et al.* [3]
	H-LompB-R1	GCACTAGCACTTGCTAAAGTACCGT			
SFGR *Candidatus* Rickettsia longicornii *sca4*	H-Lsca4-F2	AGTTCTCAGTCCAGCACAACAAC	63	885	Jiang *et al.* [3]
	H-Lsca4-R2	GCCTTTACCAGCTCATCTACTTT			
SFGR *Candidatus* Rickettsia longicornii *rrs*	H-L16S-F	TGCAAGTCGAACGGACTAATTGG	65	976	Jiang *et al.* [3]
	H-L16S-R	AATGAGGGTTGCGCTCGTTG			
SFTSV Small	S-F1	ACACAAAGACCCCCTTCATTTGGA	58	588	Liu *et al.* [10]
	S-R1	TGGAGGAGGGCCACATCCAG			
SFTSV medium	M-F1	GATGAGATGGTCCATGCTGATTCT	58	560	Liu *et al.* [10]
	M-R1	CTCATGGGGTGGAATGTCCTCAC			
SFTSV large	L-F1	ACACAGAGACGCCCAGATGAAC	60	684	Liu *et al.* [10]
	L-R1	GCCTCAAGCTCTTCCTCACTCTTCTG			
*T. orientalis* MPSP	P1	CACGCTATGTTGTCCAAGAG	53	875	Ota *et al.* [15]
	P2	TGTGAGACTCAATGCGCCTA			
*T. sinensis* MPSP	P3	CACTGCTATGTTGTCCAAGAGATATT	56	887	Liu *et al.* [8]
	P4	AATGCGCCTAAAGATAGTAGAAAAC			

### Sequence identity and phylogenetic analyses

The PCR products of the positive samples were sent to Shanghai Shenggong Co., Ltd. for sequencing. The correct gene sequences were analyzed in DNAStar and GenBank, and phylogenetic trees were constructed based on sequences obtained in this study and those previously published, using the maximum likelihood method with relative models by MEGA 7.0 software.

### Statistical analysis

Data were processed using Microsoft Excel 2007 and statistical analysis was carried out using SAS8.2 software. Numerical data were expressed as a constituent ratio (%) and positive rate (%), where the constituent ratio (%) = (number of each tick species in the same location/total number of all tick species in same location) × 100, and the positive rate (%) = number of positive samples detected for pathogens/total number of tested samples of the same species (*n*) × 100.

## Results

### Tick species survey and pathogens in ticks

A total of 2673 ticks were collected, including 1373 *Haemaphysalis longicornis* (51.37%), 651 *Ixodes persulcatus* (24.35%), 357 *Haemaphysalis japonica* (13.36%), 140 *Dermacentor silvarum* (5.24%), and 152 *Haemaphysalis concinna* (5.68%) (Table 2).

**Table 2:** Composition of tick species in 8 counties and cities of Yanbian.

Location	*Haemaphysalis longicornis*		*Ixodes persulcatus*		*Haemaphysalis japonica*		*Dermacentor silvarum*		*Haemaphysalis concinna*		Total	
	Quantity	Constituent ratio (%)	Quantity	Constituent ratio (%)	Quantity	Constituent ratio (%)	Quantity	Constituent ratio (%)	Quantity	Constituent ratio (%)	Quantity	Constituent ratio (%)
Hunchun	348	68.24	129	25.29	17	3.33	16	3.14	0	0.00	510	100.00
Tumen	37	20.22	0	0.00	120	65.57	14	7.65	12	6.56	183	100.00
Yanji	192	48.98	174	44.39	0	0.00	0	0.00	26	6.63	392	100.00
Dunhua	0	0.00	106	42.74	125	50.40	17	6.85	0	0.00	248	100.00
Helong	87	26.69	158	48.46	67	20.55	14	4.29	0	0.00	326	100.00
Longjing	426	88.38	56	11.62	0	0.00	0	0.00	0	0.00	482	100.00
Wangqing	283	84.73	23	6.89	28	8.38	0	0.00	0	0.00	334	100.00
Antu	0	0.00	5	2.53	0	0.00	79	39.90	114	57.58	198	100.00
Total	1,373	51.37	651	24.35	357	13.36	140	5.24	152	5.68	2673	100.00

After screening for pathogens by targeting different genes, we detected CRT, SFGR, SFTSV, and *T. orientalis* in *H. longicornis*, CRT, SFGR, and SFTSV in *I. persulcatus* and *H. japonica*, CRT and SFTSV in *D. silvarum*, and only SFTSV in *H. concinna*. Moreover, different CRT and SFGR genotypes were identified in *H. longicornis* and *H. japonica*, while different SFTSV genotypes were confirmed in *H. concinna*. Mixed infection with CRT and SFTSV in *I. persulcatus*, *H. japonica,* and *D. silvarum* were observed. The highest frequency of CRT/SFTSV co-infection included 13 cases (2.00%) in *I. persulcatus*, five cases (1.40%) in *H. japonica*, and one case (0.71%) in *D. silvarum*. *Theileria orientalis* was detected in *H. longicornis*, while *T. sinensis* was not detected in any ticks (Table 3).

**Table 3:** Infection of pathogens in different tick species in Yanbian, China.

Species	CRT ompA	CRT *17kDa*	SFGR *Candidatus* R. longicornii *ompA*	SFGR *Candidatus* R. longicornii *ompB*	SFGR *Candidatus* R. longicornii *sca4*	SFGR *Candidatus* R. longicornii *rrs*	SFTSV Small	SFTSV Medium	SFTSV Large	CRT + SFTSV	*T. orientalis* MPSP	*T. sinensis* MPSP
	Number of positives (%)	Number of positives (%)	Number of positives (%)	Number of positives (%)	Number of positives (%)	Number of positives (%)	Number of positives (%)	Number of positives (%)	Number of positives (%)	Number of positives (%)	Number of positives (%)	Number of positives (%)
*Haemaphysalis longicornis* (*n* = 1373)	22 (1.60)	23 (1.68)	323 (23.53)	261 (19.01)	287 (20.90)	364 (26.51)	16 (1.17)	13 (0.95)	15 (1.09)	0	13 (0.95)	0
*Ixodes persulcatus* (*n* = 651)	71 (10.91)	57 (8.76)	7 (1.08)	5 (0.77)	5 (0.77)	6 (0.92)	22 (3.38)	17 (2.61)	26 (3.99)	13 (2.00)	0	0
*Haemaphysalis japonica* (*n* = 357)	26 (7.28)	25 (7.00)	8 (2.24)	6 (1.68)	7 (1.96)	7 (1.96)	38 (10.64)	23 (6.44)	27 (7.56)	5 (1.40)	0	0
*Dermacentor silvarum* (*n* = 140)	28 (20.00)	49 (35.00)	0	0	0	0	44 (31.43)	26 (18.57)	37 (26.43)	1 (0.71)	0	0
*Haemaphysalis concinna* (*n* = 152)	0	0	0	0	0	0	17 (11.18)	11 (7.24)	19 (12.50)	0	0	0
Total (*n* = 2673)	147 (5.50)	154 (5.76)	338 (12.64)	272 (10.18)	299 (11.19)	377 (14.10)	137 (5.13)	90 (3.37)	124 (4.64)	19 (0.71)	13 (0.49)	0

### Percent identities and phylogenetic analyses

**Figure 2:**
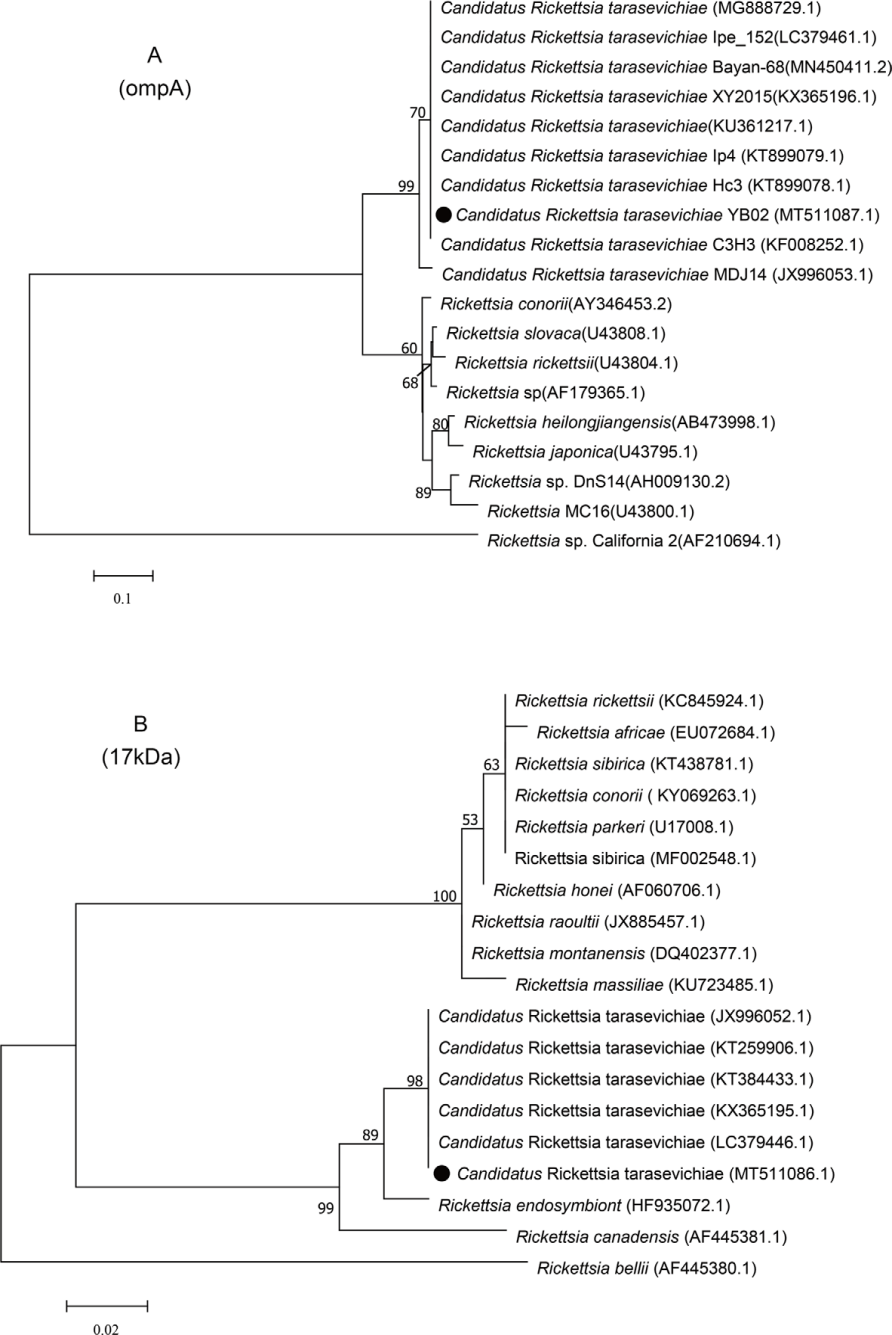
Phylogenetic trees based on the *ompA* and *17kDa* genes of CRT. The ML trees were implemented by MEGA7 with a Hasegawa–Kishino–Yano model. The numbers at the nodes represent percentage of occurrence of clades in 1000 bootstrap replications of data. The gene sequences from this study are indicated by a round shape.

Analysis of the sequence identity showed that the CRT *ompA* gene sequences generated from ticks in Yanbian YB02 strain (MT511087) shared 100% identity with the Henan Xinyang strain (KX365196), Northeast China strain (KT899079), and Heilongjiang Mudanjiang strain (JX996053). Phylogenetic analysis showed that the CRT *ompA* sequence from Yanbian clustered with the sequences mentioned above (Fig. 2). In a similar manner, the *17-kDa* gene sequence (MT511086) obtained in this study shared 99.7%–100% identities and clustered with sequences from Henan Xinyang (KX365195), Heilongjiang Mudanjiang (KT259906), and Jilin (KT384433) (Fig. 2).

**Figure 3:**
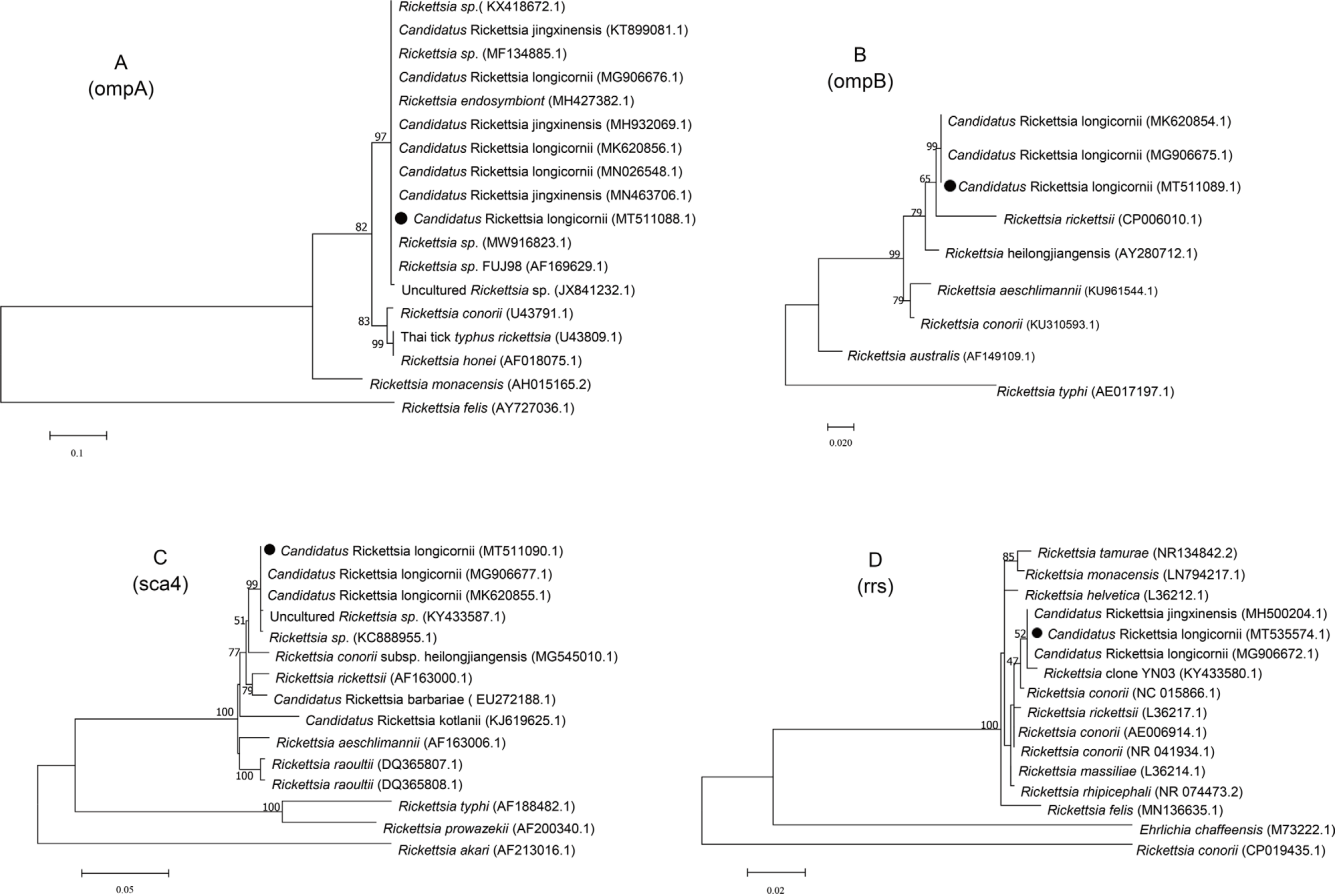
Phylogenetic trees based on the SFGR *Candidatus* Rickettsia longicornii ompA, ompB, sca4 and rrs partial sequences obtained from *Haemaphysalis longicornis*. The ML trees were implemented by MEGA7 with a Kimura 2-parameter model. The numbers at the nodes represent percentage of occurrence of clades in 1000 bootstrap replications of data. The gene sequences from this study are indicated by a round shape.

From the samples positive for SFGR *Candidatus* R. longicornii*,* four fragments of the *ompA, ompB, sca4*, and *rrs* genes were obtained by PCR amplification. Phylogenetic analysis showed that the *ompA* sequence of *Candidatus* R. longicornii (MT511088) from this study formed one cluster with the isolates from Korea (MG906676), Chinese Changchun (KT899081), and Chinese Dandong (MH427382). The *ompB* gene sequence (MT511089) was in the same clade as the Chinese HC strain (MK620854) and exhibited a close evolutionary relationship with the Korean ROK-HL727 strain (MG906675). The *rrs* gene sequence (MT535574) formed one cluster with the *Candidatus* Rickettsia jiangxiensis-related sequence (MH500204) found in *H. longicornis* in China and *Candidatus* R. longicornii ROK-HL727 strain (MG906672) in Korea. The *sca4* gene sequence (MT511090) was also closely related to the Korean *Candidatus* R. longicornii ROK-HL727 strain (MG906677.1) (Fig. 3). Sequence analysis showed that the four gene sequences (MT511088, MT511089, MT511090, and MT535574) shared 100%, 99.70%, 100%, and 95.70% identities, respectively with the corresponding fragments (MG906676, MG906675, MG906677, and MG906672) of SFGR *Candidatus* R. longicornii newly discovered in the Korean *H. longicornis* (ROK-HL727).

**Figure 4:**
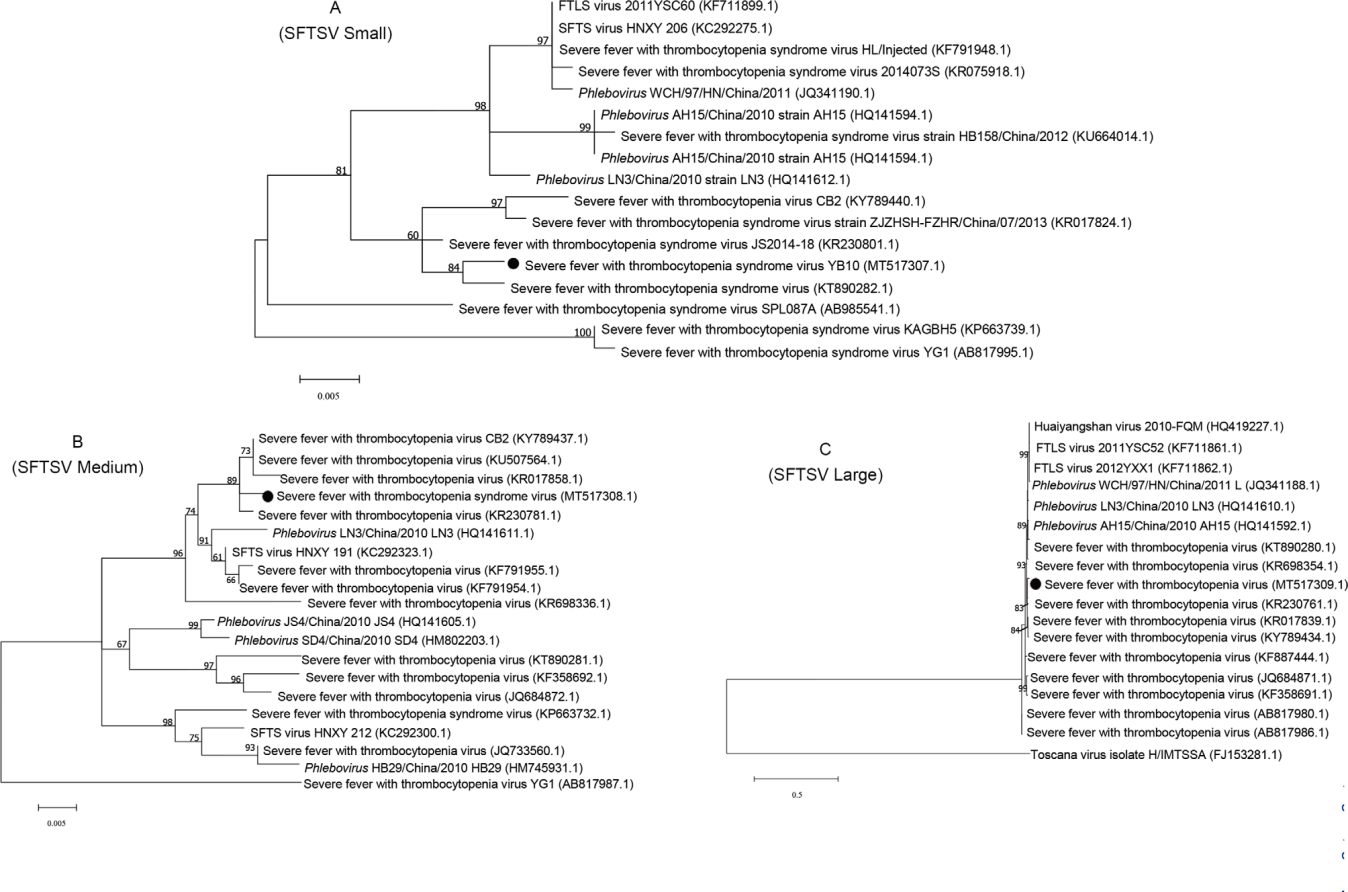
Phylogenetic tree based on the SFTSV small, medium and large partial sequences obtained from *Haemaphysalis longicornis*. The ML trees were implemented by MEGA7 with a Tamura 3-parameter model. The numbers at the nodes represent percentage of occurrence of clades in 1000 bootstrap replications of data. The gene sequences from this study are indicated by a round shape.

The SFTSV Large (MT517309), Medium (MT517308), and Small (MT517307) gene sequences were identified in this study. The nucleotide sequence identity data demonstrated that sequences of SFTSV obtained in this study shared more than 95% identity with most of the SFTSV gene sequences previously identified in China and South Korea. The phylogenetic analysis showed that the SFTSV Small gene sequence from ticks in Yanbian (MT517307) was in the same clade as the SFTSV gene sequence (KT890282) from Jilin ticks in China. The Medium gene sequence (MT517308) formed one clade with the Chinese JS2014-18 strain (KR230781), and the Large sequence was located in the same branch as the Chinese JS2014-18 strain (KR230761) and was closely related to SFTSV isolated from Zhejiang (KR017839) and South Korea (KY789434) (Fig. 4).

**Figure 5:**
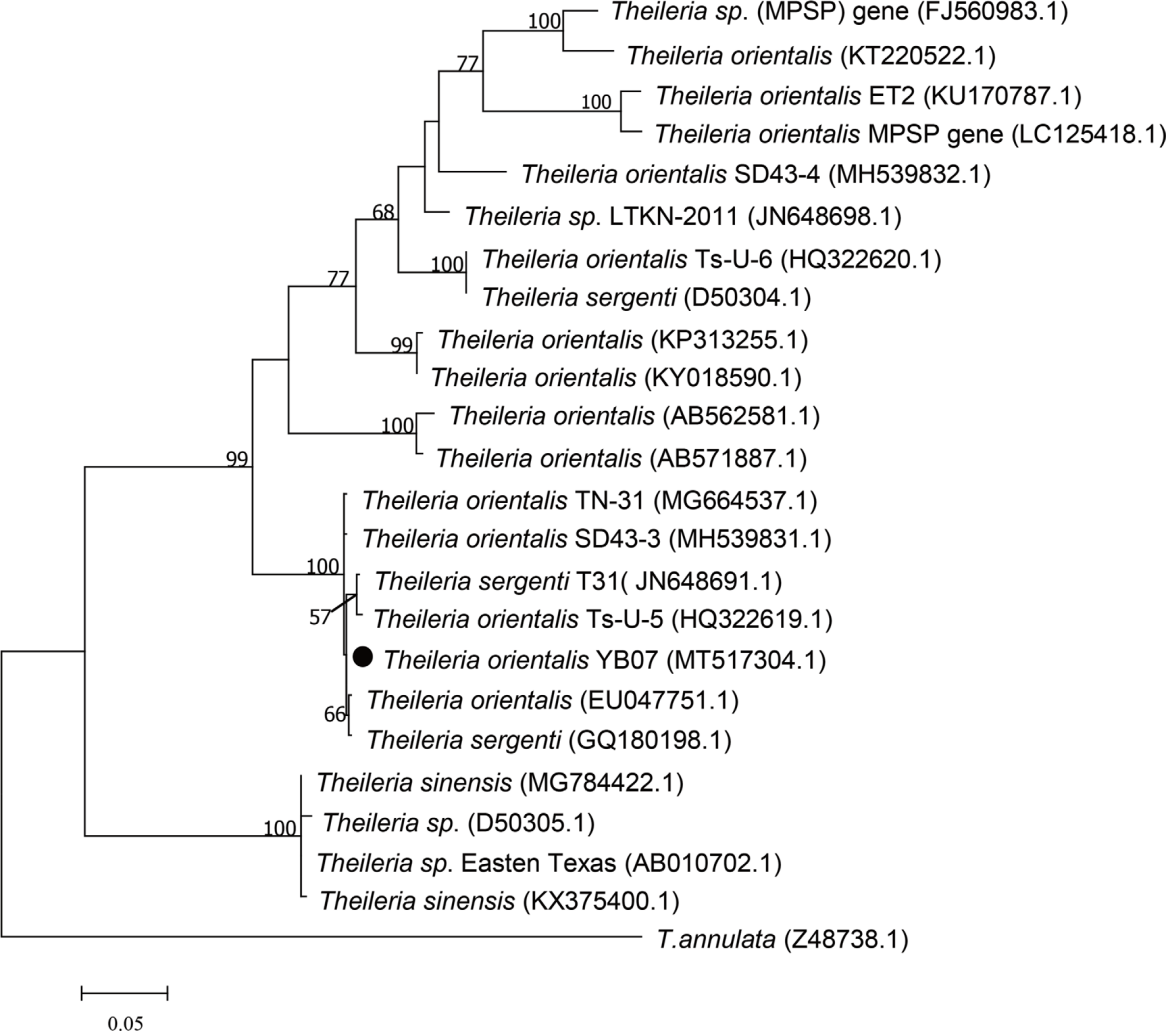
Phylogenetic tree based on the *T****.*** *orientalis* MPSP sequences obtained from *Haemaphysalis longicornis***.** The ML trees were implemented by MEGA7 with a Kimura 2-parameter model**.** The numbers at the nodes represent percentage of occurrence of clades in 1**,**000 bootstrap replications of data**.** The gene sequence from this study is indicated by a round shape**.**

The MPSP gene sequence obtained from this study (MT517304) was 99.4% identical to that of the *T. orientalis* Chongqing (MG664537) isolate. The phylogenetic analysis showed that the *T. orientalis MPSP* gene sequence in this study was classified nearer the cluster of *T. sergenti* than *T. sinensis*. The *T. orientalis* MPSP gene isolate in our study was 68.2% – 99.6% identical to the sequences of *T. orientalis* and *T. sergenti* cited in this study (Fig. 5).

## Discussion

Yanbian is located at the junction of China, North Korea, and Russia, and has a long border spanning 755.2 km. Due to strengthened ecological and environmental protection in China, the species along the border have diversified gradually. Thus, the number and species of ticks are constantly changing, and their activity is increasing. Ticks and other vectors in the border zone can freely migrate to another country through a variety of routes, which may increase the risk of infection with tick-borne diseases. In this study, 2673 ticks collected from eight counties and cities in Yanbian were classified and analyzed. Among the six identified species, *H. longicorni*s and *I. persulcatus* were the dominant tick species in Yanbian. *Haemaphysalis longicornis* is widely distributed throughout Asia and the Pacific, including China, Russia, South Korea, Japan, Australia, New Zealand, and the South Pacific islands, and is known for its strong reproductive ability and environmental adaptability. While it often parasitizes medium and large wild or domestic animals, humans are accidental hosts of *H. longicorni*s. Moreover, *H. longicornis* spreads a variety of pathogens that can affect wild animals and livestock, as well as human health [3].

Ticks can be infected with various pathogens, including viruses, bacteria, and spirochetes. Ticks can act as both vectors and hosts in the transmission of disease. At present, the main research into co-infections of tick-borne pathogens has been focused on *Borrelia burgdorferi*, *Babesia microti*, *Ehrlichia*, and *Anaplasma phagocytophilum* [26]. Previous studies have confirmed that one-third of patients with a CRT infection had neurological symptoms that differed from other SFGR infections [11], and were associated with a higher case-fatality rate during co-infection with SFTSV [20]. Thus, more attention should be paid to SFTSV transmission through both tick bites and close contact with infected cases [27]. In this study, we confirmed the occurrence of CRT/SFTSV co-infection in Yanbian ticks. *Ixodes persulcatus* is a common dominant tick species in Yanbian, and is especially widely distributed in Hunchun, Yanji, Helong, and other regions, resulting in a higher risk of infection with CRT and SFTSV *via* tick bites in these regions.

SFGR forms a long-lasting infection cycle between ticks and mammals and can also be transmitted vertically through tick eggs, making ticks the main host and vector of SFGR. In 2016, a new genotype of SFGR, *Candidatus* R. jingxinensis, was identified in *H. longicornis* in Northeast China by Liu *et al.* [9]. Jiang *et al.* [3] reported the new SFGR genotype, *Candidatus* R. longicornii, in *H. longicornis* in Korea. The new genotypes of SFGR, *Candidatus* R. longicornii *ompA, ompB, sca4*, *and rrs* genes, were all detected in *H. longicornis*, *I. persulcatus*, and *H. japonica* collected in this study. The SFGR *Candidatus* R. longicornii gene sequences detected in our study showed high identity with the related gene sequences newly discovered in South Korea (ROK-HL727) and the sequences of *ompA* and *ompB* genes belonging to an unknown SFGR genotype found in *H. longicornis* from Dandong, China (border between China and North Korea). The results of our study suggested that the new SFGR *Candidatus* R. longicornii genotypes are widely distributed throughout the border between China and North Korea. Although there have been no reports of infections caused by the new SFGR genotype, the *ompA* gene sequence of *Candidatus* R. longicornii found in Yanbian was highly identical to an unknown species of *Rickettsia* detected in mouse spleen tissue in South Korea [3]. These findings indicate that the *Candidatus* R. longicornii identified in this study was likely to be infectious in mammalian hosts and even in humans. Therefore, it is necessary to strengthen the surveillance for the SFGR *Candidatus* R. longicornii infection in ticks and relevant hosts in the border of China, North Korea, and Russia, as well as other areas with a concentrated distribution of *H. longicornis*, in order to prevent cross-border transmission and an epidemic of tick-borne diseases affecting human health and animal husbandry [3].

SFTS is a novel infectious disease that was first discovered in China [5, 6, 12]. SFTSV was first isolated from *H. longicornis* in Korea [22]. The SFTSV Small, Medium, and Large gene sequence analysis indicated that sequences from the present study had more than 95% identity with the SFTSV gene sequences found in South Korea. Phylogenetic analysis showed that the SFTSV Small, Medium, and Large gene sequences were in the same clade as the isolates from Jilin and Jiangsu, and were closely related to SFTSV isolated from Zhejiang and South Korea. This may be related to the tick-parasitized migratory birds from the eastern part of China or the transmission by migratory birds infected with SFTSV. A previous study suggested that migratory birds may play an important role in the spread of SFTSV [23]. The abovementioned results suggest that the border area of China, North Korea, and Russia may be a key region for tick-borne SFTSV, and should be considered in the prevention and control of imported infectious diseases.

*Theileria orientalis* is a protozoan parasite that infects cattle and buffalo and is generally transmitted by ticks of the genus *Haemaphysalis* [7]. *Theileria sinensis* named by Chinese scholars was originally isolated from naturally infected cattle by Bai *et al.* in Gansu Province [1]. *Theileria orientalis* is transmitted by *H. longicornis*, *H. concinna*, and *H. japonica* ticks. However, the tick species differ among regions; for example, *H. japonica* is the main vector of Oriental Taylor disease in Russia, followed by *H. concinna*. *Haemaphysalis concinna* is also the main vector in Korea, whereas *H. longicornis* is the main vector in China and Japan. In this investigation, we detected *T. orientalis* in *H. longicornis* but failed to detect *T. sinensis* in any ticks. The non-detection *of T. sinensis* may be attributed to the relatively smaller collection area and small numbers of its vector, *H. japonica*.

In this study, some regional endemic tick-borne pathogens were detected, such as CRT, SFGR, SFTSV, and *Theileria*, but whether there are other tick-borne pathogens remains to be studied. The phylogenetic analysis of tick-borne pathogens was mainly described and analyzed using the epidemic strains in China and the strains in Korea adjacent to Yanbian. The results of our study determined the epidemic trend of tick-borne pathogens in the border zone among China, Russia and North Korea.

## Conclusions

*Haemaphysalis longicornis* and *I. persulcatus* are the dominant tick species in Yanbian, China. Four pathogens (CRT, SFGR, SFTSV and *T. orientalis*) were detected in the tick species collected in this study, and CRT/SFTSV co-infection was also identified in *I. persulcatus* and *H. japonica*. Moreover, this study provides the first evidence of the SFGR genotypes *Candidatus* R. longicornii *ompA, ompB, sca4*, and *rrs* in *H. longicornis* in Yanbian, China. In addition, *T. orientalis* was detected in *H. longicornis*. These findings provide epidemiological data to support the prevention and control of ticks and tick-borne diseases in the border region of China, North Korea, and Russia.

## References

[1] Bai Q, Liu Y, Yin H, Zhao QZ, Liu DK, Ren JX. 2002. *Theileria Sinensis* sp nov: A new species of bovine *Theileria* – classical taxonomic studies. Acta Veterinaria et Zootechnica Sinica, 1, 73–77.

[2] Horak IG, Camicas JL, Keirans JE. 2002. The Argasidae, Ixodidae and Nuttalliellidae (Acari: Ixodida): a world list of valid tick names. Experimental & Applied Acarology, 28, 27–54. 14570115 10.1023/a:1025381712339

[3] Jiang J, An H, Lee JS, O’Guinn ML, Kim HC, Chong ST, Zhang Y, Song D, Burrus RG, Bao Y, Klein TA, Richards AL. 2018. Molecular characterization of *Haemaphysalis longicornis-borne rickettsiae*, Republic of Korea and China. Ticks and Tick-Borne Diseases, 9(6), 1606–1613. 30100386 10.1016/j.ttbdis.2018.07.013

[4] Jia N, Jiang JF, Huo QB, Jiang BG, Cao WC. 2013. *Rickettsia sibirica subspecies sibirica* BJ-90 as a cause of human disease. New England Journal of Medicine, 369(12), 1176–1178. 24047079 10.1056/NEJMc1303625

[5] Jiang XL, Wang XJ, Li JD, Ding SJ, Zhang QF, Qu J. 2012. Isolation, identification and characterization of SFTS bunyavirus from ticks collected on the surface of domestic animals. Bing Du Xue Bao, 28, 252–257. 22764528

[6] Jing JW, Zhi XL, Xiu LW, Jing Y, Jian L, Hua BT. 2019. Research progress of severe fever with thrombocytopenia syndrome and its etiology. Tianjin Medical Journal, 47, 220–224.

[7] Kawazu S, Sugimoto C, Kamio T, Fujisaki K. 1992. Antigenic differences between Japanese *Theileria sergenti* and other benign *Theileria* species of cattle from Australia (*T. buffeli*) and Britain (*T. orientalis*). Parasitology Research, 78(2), 130–135. 1557325 10.1007/BF00931654

[8] Liu A, Guan G, Liu Z, Liu J, Leblanc N, Li Y, Gao J, Ma M, Niu Q, Ren Q, Bai Q, Yin H, Luo J. 2010. Detecting and differentiating *Theileria sergenti* and *Theileria sinensis* in cattle and yaks by PCR based on major piroplasm surface protein (MPSP). Experimental Parasitology, 126(4), 476–481. 20685208 10.1016/j.exppara.2010.05.024

[9] Liu H, Li Q, Zhang X, Li Z, Wang Z, Song M, Wei F, Wang S, Liu Q. 2016. Characterization of rickettsiae in ticks in northeastern China. Parasites & Vectors, 9(1), 498. 27623998 10.1186/s13071-016-1764-2 PMC5022169

[10] Liu H, Li Z, Wang Z, He B, Wang S, Wei F, Tu C, Liu Q. 2016. The first molecular evidence of severe fever with thrombocytopenia syndrome virus in ticks in Jilin, Northeastern China. Ticks and Tick-Borne Diseases, 7(6), 1280–1283. 27460903 10.1016/j.ttbdis.2016.06.007

[11] Liu W, Li H, Lu QB, Cui N, Yang ZD, Hu JG, Fan YD, Guo CT, Li XK, Wang YW, Liu K, Zhang XA, Yuan L, Zhao PY, Qin SL, Cao WC. 2016. *Candidatus Rickettsia tarasevichiae* Infection in Eastern Central China: A case series. Annals of Internal Medicine, 164(10), 641–648. 27019406 10.7326/M15-2572

[12] Liu Y, Huang XY, Du YH, Wang HF, Xu BL. 2012. Survey on ticks and detection of new bunyavirus in some vectors in the endemic areas of fever, thrombocytopenia and leukopenia syndrome (FTLS) in Henan province. Zhong Hua Yu Fang Yi Xue Za Zhi, 46, 500–504. 22943894

[13] Lu BL, Wu HY. 2003. Classification and identification of important medical insects of China. Henan Science and Technology Publishing House, pp. 661–665.

[14] Oliver JH. 1989. Biology and systematics of ticks (Acari: Ixodida). Annual Review of Ecology and Systematics, 20, 397–430.

[15] Ota N, Mizuno D, Kuboki N, Igarashi I, Nakamura Y, Yamashina H, Hanzaike T, Fujii K, Onoe S, Hata H, Kondo S, Matsui S, Koga M, Matsumoto K, Inokuma H, Yokoyama N. 2009. Epidemiological survey of *Theileria orientalis* infection in grazing cattle in the eastern part of Hokkaido, Japan. Journal of Veterinary Medical Science, 71(7), 937–944. 19652482 10.1292/jvms.71.937

[16] Parola P, Paddock CD, Raoult D. 2005. Tick-borne rickettsioses around the world: emerging diseases challenging old concepts. Clinical Microbiology Reviews, 18, 719–756. 16223955 10.1128/CMR.18.4.719-756.2005 PMC1265907

[17] Peñalver E, Arillo A, Delclòs X. 2018. Publisher Correction: Ticks parasitised feathered dinosaurs as revealed by Cretaceous amber assemblages. Nature Communications, 9, 472. 10.1038/s41467-018-02913-w PMC5790008 29382823

[18] Schötta AM, Wijnveld M, Stockinger H, Stanek G. 2017. Approaches for reverse line blot-based detection of microbial pathogens in *Ixodes ricinus* ticks collected in Austria and impact of the chosen method. Applied and Environmental Microbiology, 83(13), e00489-17. 28455331 10.1128/AEM.00489-17 PMC5478998

[19] Wei L. 2017. Emerging tick borne agents in China. Infectious Disease Information, 30, 11–14.

[20] Yadi F, Jian GH, Xiao MC, Chen TG, Ying W, Xiao AZ. 2016. Co-infection of spotted fever group rickettsiae and severe fever with thrombocytopenia syndrome virus in ticks in eastern central China. Acta Parasitologica et Medica Entomologica Sinica, 23, 86–90.

[21] Yamaguti N, Tipton VJ, Keegan HL. 1971. Ticks of Japan, Korea, and the Ryukyu Islands Brigham Young University Science Bulletin. Biological Series, 15, 226.

[22] Yun SM, Song BG, Choi W, Roh JY, Lee YJ, Park WI, Han MG, Ju YR, Lee WJ. 2016. First isolation of severe fever with thrombocytopenia syndrome virus from *Haemaphysalis longicornis* ticks collected in severe fever with thrombocytopenia syndrome outbreak areas in the Republic of Korea. Vector Borne and Zoonotic Diseases, 16(1), 66–70. 26745758 10.1089/vbz.2015.1832 PMC4742983

[23] Yun Y, Heo ST, Kim G, Hewson R, Kim H, Park D, Cho NH, Oh WS, Ryu SY, Kwon KT, Medlock JM, Lee KH. 2015. Phylogenetic analysis of severe fever with thrombocytopenia syndrome virus in South Korea and migratory bird routes between China, South Korea, and Japan. American Journal of Tropical Medicine and Hygiene, 93(3), 468–474. 26033016 10.4269/ajtmh.15-0047 PMC4559681

[24] Yu Z, Wang H, Wang T, Sun W, Yang X, Liu J. 2015. Tick-borne pathogens and the vector potential of ticks in China. Parasites & Vectors, 8, 24. 25586007 10.1186/s13071-014-0628-x PMC4300027

[25] Ze C, Tinghuan W. 2017. The world list of ticks. 2. Ixodinae (Acari: Ixodida: Ixodidae). Chinese Journal of Parasitology and Parasitic Diseases, 35, 371–381.

[26] Zhang H, Sun Y, Jiang H, Huo X. 2017. Prevalence of severe febrile and thrombocytopenic syndrome virus, *Anaplasma* spp. and *Babesia microti* in hard ticks (Acari: Ixodidae) from Jiaodong Peninsula, Shandong Province. Vector Borne Zoonotic Diseases, 17(2), 134–140. 28048951 10.1089/vbz.2016.1978

[27] Zhou XC, Fei W, Bang QQ, En FC, Jia BW. 2014. Investigation and disposal on the first cluster outbreak of person to person transmission of severe fever with thrombocytopenia syndrome in southern Anhui Province. Chinese Journal of Disease Control & Prevention, 18, 1055–1058.

